# IFN gamma regulates proliferation and neuronal differentiation by STAT1 in adult SVZ niche

**DOI:** 10.3389/fncel.2015.00270

**Published:** 2015-07-13

**Authors:** Leticia Pereira, Rebeca Medina, Miguel Baena, Anna M. Planas, Esther Pozas

**Affiliations:** ^1^Unit of Brain Ischemia, Institut d’Investigacions Biomèdiques August Pi i Sunyer (IDIBAPS)Barcelona, Spain; ^2^Department of Brain Ischemia and Neurodegeneration, Institute of Biomedical Research of Barcelona, Consejo Superior de Investigaciones Científicas (CSIC)Barcelona, Spain

**Keywords:** IFNγ, neurogenesis, STAT1, SVZ, differentiation

## Abstract

The adult subventricular zone (SVZ) is the main neurogenic niche in normal adult brains of mice and rats. Interferon gamma (IFNγ) has somewhat controversially been associated with SVZ progenitor proliferation and neurogenesis. The *in vivo* involvement of IFNγ in the physiology of the adult SVZ niche is not fully understood and its intracellular mediators are unknown. Here we show that IFNγ, through activation of its canonical signal transducer and activator of transcription 1 (STAT1) pathway, acts specifically on Nestin+ progenitors by decreasing both progenitor proliferation and the number of cycling cells. In addition, IFNγ increases the number of neuroblasts generated without shifting glial fate determination. The final result is deficient recruitment of newborn neurons to the olfactory bulb (OB), indicating that IFNγ-induced stimulation of neuronal differentiation does not compensate for its antiproliferative effect. We conclude that IFNγ signaling via STAT1 in the SVZ acts dually as an antiproliferative and proneurogenic factor, and thereby regulates neurogenesis in normal adult brains.

## Introduction

The subventricular zone (SVZ) of the lateral ventricle (LV) is the main neurogenic area in the adult murine brain. Neural stem cells [NSCs, glial fibrillary acidic protein, (GFAP+) cells and Type B cells] give rise to transit amplifying dividing cells (TAPs) or Type C cells (GFAP-, EGFR+, Ki67+ and Mash1+) that mainly generate neuroblasts (Type A cells, PSA-NCAM+, DCX+ and TUBB3+), which migrate through the rostral migratory pathway (RMS) to reach the olfactory bulb (OB; Lois and Alvarez-Buylla, 1994; Doetsch and Alvarez-Buylla, 1996; Doetsch et al., 1997; Merkle et al., 2004). This process can be regulated by physiological and pathological conditions, with both the integration of extracellular signaling activation and the presence of some intrinsic mechanisms leading to cell fate determination and lineage differentiation (Guillemot, 2007; Lim et al., 2009).

Recently, it has been reported that the immune system plays a key role in regulating the NSC population through the actions of chemokines and cytokines. Low levels of cytokines are in circulation under physiological conditions, and it has been suggested that the proper balance between pro- and anti-inflammatory effects results in a equilibrate neurogenesis; while neurogenesis is impaired under inflammatory conditions (Ekdahl et al., 2003; Monje et al., 2003; Liu et al., 2007; Li et al., 2010; Pérez-Asensio et al., 2013; Pereira et al., 2015). Some pro-inflammatory cytokines, such as interleukin-6 (IL-6) and interleukin-1 (IL-1), are anti-neurogenic (Vallières et al., 2002; Monje et al., 2003; Koo and Duman, 2008). Recently, we showed that the anti-inflammatory cytokine IL-10 plays an important role in normal SVZ regulation through the extracellular-signal-regulated kinases (ERK) and STAT3 intracellular intermediaries, by which it regulates the cell cycle activity of SVZ progenitors and maintains their undifferentiated stage (Pérez-Asensio et al., 2013; Pereira et al., 2015). Other, anti-inflammatory cytokines, such as IL-4, can induce microglial secretion of insulin-like growth factor-1 (IGF-1), thereby promoting neurogenesis (Butovsky et al., 2006).

Interferon gamma (IFNγ) is a pro-inflammatory cytokine involved in the pathology of the neuroinflammatory response that is mostly released from activated microglia (Chavarria and Alcocer-Varela, 2004; Na et al., 2014; Singhal et al., 2014). The cerebrospinal fluid (CSF) and blood of healthy animals contain low concentrations of IFNγ, and IFNγ messenger and IFNγ receptor (IFNGR) are normally expressed in the neurogenic areas of the brain during development and adulthood (Li et al., 2010). Some *in vitro* evidences indicate that IFNγ induces neuronal differentiation in a range of neural cultures (Wong et al., 2004; Zahir et al., 2009; Li et al., 2010; Turbic et al., 2011; Walter et al., 2011). In addition, (Li et al., 2010) described how, in normal brain in the chronic absence of IFNγ, *in vivo* proliferation of SVZ progenitors is increases, as does the number of new neurons incorporated into the OB of IFNγ-deficient animals. Those authors also reported that *in vitro* SVZ progenitors from IFNγ-deficient animals formed more neurospheres as well as both neuroblasts and oligodendrocytes (Li et al., 2010). All these *in vitro* observations in different progenitor cultures lead to some controversy related to the role of IFNγ in SVZ neuronal differentiation. Nonetheless, no data have been reported regarding the involvement of IFNγ in neuronal differentiation *in vivo*.

In the present paper, we establish the physiological role of IFNγ in the regulation of both progenitor proliferation and neuronal differentiation in the adult SVZ niche *in vivo*; and we propose signal transducer and activator of transcription 1 (STAT1) as its intracellular mediator. We demonstrate how IFNγ exhibits potent antiproliferative activity while promoting neuronal differentiation of SVZ progenitors. However, the final result is impaired neurogenesis, since the antiproliferative activity exceeds the pro-neurogenic activity; and the activation of neurogenesis cannot compensate for the robust reduction in progenitor numbers. Moreover, the study reveals STAT1 as the intracellular factor that triggers IFNγ action in the adult SVZ niche.

## Materials and Methods

### Recombinant Proteins, Reagents and Animals

IFNγ was purchased from Prepotech (Rocky Hill, NJ, USA) and AG490 was from Merck-Millipore (Darmstadt, Germany). Rats (Wistar) were obtained from Charles River (Lyon, France) and STAT1 knockout (KO) mice (129S6/SvEv-Stat1tm1Rds), generated by Durbin et al. (1996), and corresponding wild type mice in the 129S6/SvEv background were originally purchased from Taconic and were maintained at the animal house of the School of Medicine (University of Barcelona, Barcelona, Spain). All animals were male and age-matched. Animals work was carried out in accordance with the European Community Council Directives on animal welfare and according to the Comité Ético de Experimentación Animal (CEEA) of the University of Barcelona. Every effort was made to minimize animal suffering.

### Primary Cultures

Cell cultures were performed from postnatal brains of rats (P7-P9) as previously described (Pérez-Asensio et al., 2013). Briefly, for dissociated cultures the SVZ was gently microdissected mildly trypsinized and platted in poly-L-lysine coated plates and cultured in Dulbecco’s Modified Eagle Medium: Nutrient Mixture F-12 (DMEM/F12) supplemented with B27 (Life Technologies, Paisley, UK). For neurospheres assay SVZ isolated cells cultured in the presence of 10 ng/ml bFGF and 20 ng/ml of epidermal growth factor (EGF) in DMEM/F12 medium plus supplements (Life Technologies); for differentiation studies were performed by mechanical dissociation of neurospheres from passage two in DMEM/F12 medium with B27 during 7 days. SVZ explants were embedded in Matrigel (BD-Bioscience) and cell migration was evaluated as described before Pérez-Asensio et al. (2013). Cells were cultured either in the presence or the absence of IFNγ (50 ng/ml). Briefly, for viability studies living cultures were stained with 10 μg Propidium Iodide (PI) to specifically label dead cells and all nuclei were stained with Hoechst. Proliferative studies were analyzed after a 4 h pulse of Bromo-deoxy-Uridine (BrdU, 10 μM; Sigma-Aldrich, St. Louis, MO, USA) in each time studied. Cell death and proliferation were represented as percentage of the total number of cells.

### Biochemistry

Western blotting of primary cultures and tissue samples were processed as previously described (Pérez-Asensio et al., 2013). Membranes were then incubated with the following antibodies: Phosphorylated STAT1, STAT3, Janus kinase 1 (JAK1) and JAK2 (all from Cell Signaling Technology, 1:1000); total STAT-1 (BD transduction, 1:1000), total STAT-3 (BD Bioscience, 1:1000); and Tubulin (Sigma, 1:50,000) or Actin (Sigma, 1:50,000) as loading controls.

### Immunofluorescence

For immunoflurescence see procedure described in Pérez-Asensio et al. (2013). The list and dilution of primary antibodies was: pSTAT1_tyr701_(1:500), BIII-Tubulin (1:1000), Doublecortin (DCX; either Cell Signaling Technology 1:1000 or Sta. Cruz Biotechnology, Santa Cruz, USA 1:500), polysialylated neuronal cell adhesion molecule (PSA-NCAM; 1:500, Merck-Millipore), Nestin (1:200, either from Merck-Millipore or BD Bioscience San Jose, USA), Ki67 (Leica Microsystems, Wetzlar, Germany 1:1000), BrdU (AbCam, 1:400), oligodendrocyte transcription factor (Olig2; Millipore, 1:400), GFAP (DAKO, 1:2000). Staining on cell cultures was visualized with a Leica CTR400-DMI400B inverted microscope or Leica DM5500Q confocal microscope. Photographs were taken by a DFC300FX camera from Leica. Quantification was carried out using Leica Application Suite (LAS-Leica) or ImageJ (NIH) software. The number of positive cells for each experimental assay was expressed as percentage of the total number and the intensity of pSTAT1_tyr701_ immunoreactivity was also evaluated in certain experiments using ImageJ software.

### Intracerebroventricular (ICV) IFNγ Infusion in the Mouse Brain

The administration of the IFNγ (50 ng/ml) at a low flow rate of 0.5 μl/h during 7 days in the third ventricle was carried out by continuous infusion with an Alzet® osmotic minipump (model 1007D) and Alzet® Brain Infusion Kit 3 (DURECT Corporation, Cupertino, CA, USA) as previously described (Pérez-Asensio et al., 2013). The delivery of saline as the vehicle or IFNγ to the third ventricle was achieved by inserting the cannula 1.7 mm depth from the brain surface at −0.1 mm posterior, and 0.6 lateral coordinates (Franklin and Paxinos, 2008), after exposing Bregma. The contralateral hemisphere (left) was always considered for histological analysis and some ipsilateral hemispheres were used for biochemical studies.

### Histology

See methodology in Pérez-Asensio et al. (2013), briefly: mice (8 weeks) were perfused with four percent paraformaldehyde and cryoprotected in 30% sucrose. Coronal sections (16 μm) were obtained and were collected in eight consecutive slice series. In all animals, the left (contralateral) hemisphere was analyzed on histological sections.

Routinely, the total number of cells in dorsal SVZ was counted per section after TO-PRO3 staining (Invitrogen). Cell death was evaluated by the pattern of cleaved Spectrin by elecrophoresis and Western blotting and cleaved Caspase three immunostaining on sections (Cell Signaling, 1:150). After immunofluorescence analysis (see protocol above), brain sections were scanned and evaluated under a Leica DM5500Q confocal microscope. At least four consecutive sections from the same slice were evaluated for each staining and the number of positive cells for each section was counted after a Z projection by ImageJ or LAS (Leica application suite) software. For the SVZ analysis, pictures were taken between +1.10 and +0.38 mm from Bregma.

### Statistical Analysis

Statistical analyses of differences between group means were performed using the two-tailed Student’s *t*-tests. In each case, *n* indicates the number of independent cultures or mice used. Statistical significance was considered always if *p* < 0.05.

## Results

### IFNγ Activates STAT1 Phosphorylation by JAK2 in Nestin+ SVZ Progenitors

**Figure 1:**
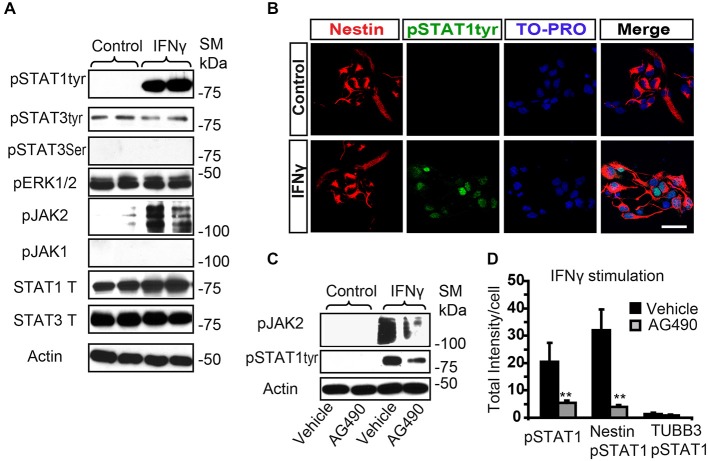
**IFNγ increases tyrosine STAT1 phosphorylation in Nestin+ SVZ progenitors. (A)** Phosphorylation of STAT1 at Tyr701 and JAK2 were induced 15 min after IFNγ (50 ng/mL) addition in primary SVZ cultures. Stimulation of pSTAT3_Tyr701_, pSTAT3_Ser727_, and JAK1 were not observed (*n* = 6 per time point). Total STAT1 and STAT3 were unchanged and actin was the loading control. **(B)** Double-immunofluorescences staining showed that STAT1_Tyr701_ immunoreactivity was absent in the control culture. After IFNγ stimulation STAT1_Tyr701_ (green) was increased exclusively in Nestin+ progenitors (red; *n* = 4), TO-PRO (blue) labeled all nuclei. **(C)** The presence of the AG490 STAT1 inhibitor reduced STAT1 phosphorylation (*n* = 4 per time point). Actin was the loading control. **(D)** Histogram represents immunoreactivity signal intensity of STAT1_Tyr701_ per Nestin+ cell after IFNγ stimulation in the presence or absence of AG490 inhibitor. After IFNγ stimulation, phosphorylation of STAT1S_Tyr701_ was increased in Nestin+ progenitors and the presence of the JAK2 phosphorylation inhibitor AG490 impeded STAT1 phosphorylation (*n* = 4). Scale bar: **(B)** 30 μm. Data are represented as mean ± SEM. **p ≤ 0.01.

The conventional signaling pathway activated by IFNγ involves sequential phosphorylation of the tyrosine residues of the JAK and the STAT proteins, as a primary gene expression regulatory mechanism (Li, 2008). The IFNGR and other surface receptors, such as complement receptor 2, which can translate cell IFNγ activity, have previously been reported in adult SVZ progenitors (Li et al., 2010; Moriyama et al., 2011). We aimed here to evaluate the signaling activity induced by IFNγ in postnatal SVZ progenitors. Stimulation assays with IFNγ in SVZ-dissociated cultures showed rapid phosphorylation of STAT1 at Tyr701 (pSTAT1_Tyr701_); while STAT3 was not stimulated at Tyr 705 (Figure 1A). Phosphorylation of Ser 727 was not induced in either STAT1 or STAT3 (pSTAT3_ser727_) (Figure 1A, and data not shown). In addition, its regular mediator, JAK2, was activated; but JAK1 was not (Figure 1A). Double immunofluorescence analysis demonstrated that pSTAT1_Tyr701_ was absent in the control situation; whereas after IFNγ stimulation, phosphorylation of STAT1 at Tyr 701 was specifically detected in the nuclei of Nestin+ progenitors (Figure 1B). The addition of the JAK2 inhibitor AG490 diminished the STAT1 phosphorylation (pSTAT1_Tyr701_) as assessed by Western blot (Figure 1C), and it severely reduced the intensity of the immunoreactivity of phosphorylated STAT1 after IFNγ stimulation on Nestin+ progenitors (Figures 1B,D).

### IFNγ Activity Inhibits Proliferation of Progenitors while Increasing Neuroblasts in SVZ-derived Postnatal Cultures

**Figure 2:**
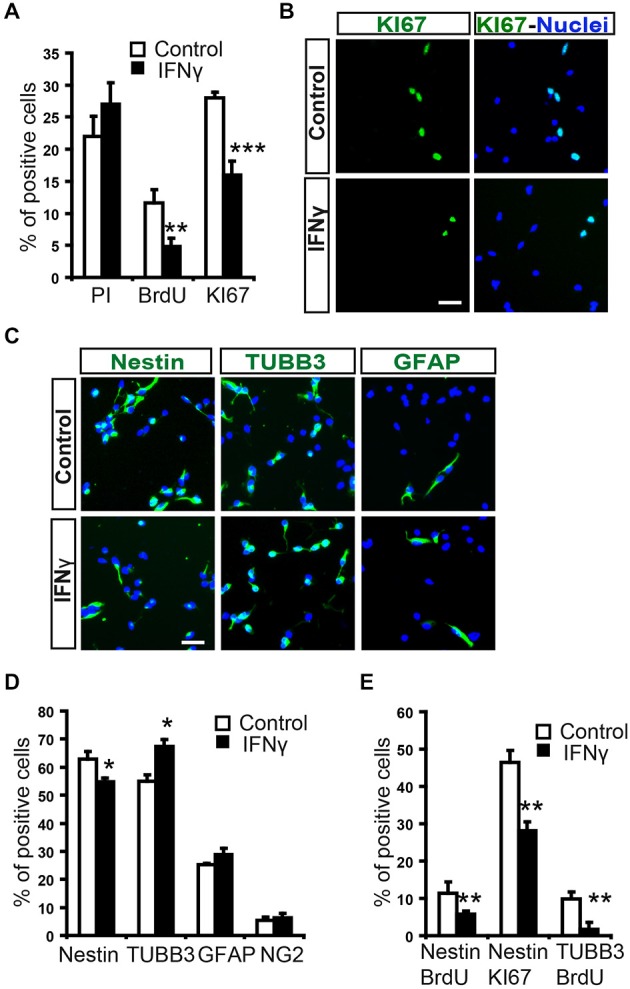
**In SVZ neural progenitors, IFNγ inhibits cell proliferation, while increasing neuroblast numbers. (A)** Histograms showing percentage cell viability (IP), proliferation (BrdU) and number of cells in active cycle (Ki67) in the presence or absence of IFNγ (50 ng/ml) in SVZ primary cultures. IFNγ did not alter cell survival, but reduced cell proliferation (BrdU+) and the number of Ki67+ cells (*n* = 7). **(B)** Immunofluorescence pictures of primary SVZ cultures illustrating the reduction of cells in active cell cycle (KI67+) after IFNγ treatment (*n* = 7). Hoechst (blue) stained all nuclei. **(C)** Immunofluorescence pictures of SVZ primary cultures illustrating the decrease of Nestin+ progenitors (red) and the increase in neuroblasts (TUBB3+, red) after IFNγ treatment for 4 days (*n* = 6). The number of GFAP+ cells remained unchanged. Hoechst (blue) stained all nuclei. **(D)** Graph summarizing the presence of cellular markers after IFNγ treatment. The numbers of TUBB3+ cells increased, while Nestin+ cells decreased in the presence of IFNγ (as percentage of total cells). Glial markers such a GFAP and Olig2 were unchanged (*n* = 6). **(E)** IFNγ cultures exhibited a consistent decrease in the number of Nestin/BrdU+ and TUBB3+/BrdU+ cells (*n* = 6), and a lower proportion of cells which expressed Nestin were in active cell cycle (Ki67+, *n* = 6). Scale bar **(B, C)**: 30 μm. Data are represented as mean ± SEM. *p < 0.05; **p ≤ 0.01; ***p ≤ 0.001.

**Figure 3:**
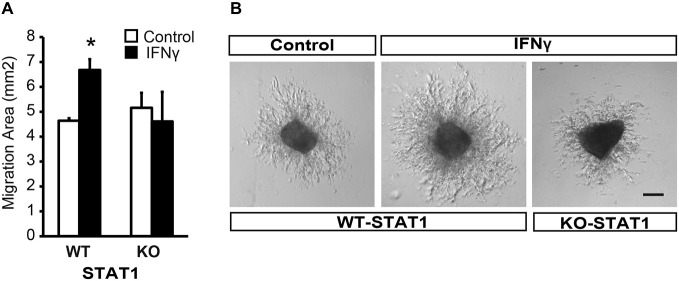
**IFNγ promotes migration from SVZ explants via STAT1. (A)** Brightfield pictures showing that IFNγ increases the migration area of progenitors exiting SVZ postnatal explants and the absence of STAT1 reverted IFNγ effects (*n* = 4). **(B)** Histograms showing the ratio of migration area of proximal (P) vs. distal(D) regions of the explants, in WT and KO samples, in the presence of IFNγ (*n* = 5). Scale bars = 100 μm. Data are represented as mean ± SEM. *p ≤ 0.05.

To evaluate the direct effect of IFNγ on SVZ progenitors, we grew monolayer primary cultures of dissociated cells from whole postnatal SVZ; as described previously (Pérez-Asensio et al., 2013). The cultures consisted of a heterogeneous cell population in which proliferation and differentiation occurred (Pérez-Asensio et al., 2013). The presence of IFNγ in the culture did not affect cell viability (PI incorporation; Figure 2A); however, cells in proliferation (BrdU+ cells; 11.6 ± 2.1% in controls vs. 4.8 ± 1.2%) or active cell cycle (Ki67+ cells; 28.1 ± 0.9% in controls vs. 16.3 ± 2.1%) were significantly decreased (Figures 2A,B). This indicates an effect of the cytokine on cell proliferation, as previously reported for NSC cultures (Ben-Hur et al., 2003; Wong et al., 2004; Li et al., 2010). Furthermore, phenotype analysis in monolayer cultures by immunofluorescence (Figure 2C) showed that the percentage of Nestin+ cells significantly decreased (62.8 ± 2.7% in controls vs. 54.8 ± 2.9%) in the presence of IFNγ; whereas the percentage of neuroblasts increased (TUBB3+ cells; 55.0 ± 2.4% in controls vs. 67.4 ± 2.5%; Figure 2D). In addition, this cytokine decreased the proportion of Nestin+ progenitors and TUBB3+ cells that incorporate BrdU (11.3 ± 3.1% in controls vs. 5.8 ± 0.9%, and 9.8 ± 1.9 1.95% in controls vs. 1.8 ± 1.3% respectively), as well as the proportion of progenitors (Nestin+) that were in active cell cycle (KI67+; 46.5 ± 3.2% in controls vs. 28.1 ± 2.4%). This suggests a more pronounced differentiation step in the presence of IFNγ (Figure 2E). The proportion of glial progenitors (either Olig2+ or GFAP+ cells) was unchanged after IFNγ treatment (Figure 2D). In order to further explore the effects of IFNγ on neuronal differentiation, SVZ explants were exposed to IFNγ. The results showed that the area of migrating cells moving away from the core of the explants was considerably increased by IFNγ, indicating a more pronounced maturation of progenitors in the presence of this cytokine (Figures 3A,B). This observation could also be explained by impaired cell migration. To rule out this possibility, we performed a migration assay where SVZ explants were confronted by a source of IFNγ (soaked beads). The IFNγ did not induce chemoattractive effects in the SVZ progenitor cultures (data not shown).

Furthermore, and in order to evaluate whether STAT1 was the intracellular mediator of IFNγ, we analyzed the response of SVZ explants from STAT1-deficient mice (STAT1-KO; Durbin et al., 1996). In the absence of STAT1 when IFNγ was present, the area of migrating cells was similar to that in the control situation (Figure 3B), indicating that STAT1 is needed for the effect of IFNγ on SVZ cells.

Altogether, these results indicate that IFNγ is a regulator of both cell proliferation and neuronal differentiation through STAT1, and as a consequence, it modulates the final neurogenesis outcome of SVZ progenitors.

### Through STAT1 Activation, IFNγ Inhibits Neurosphere Formation and Enhances Neuronal Differentiation in NSC Cultures

**Figure 4:**
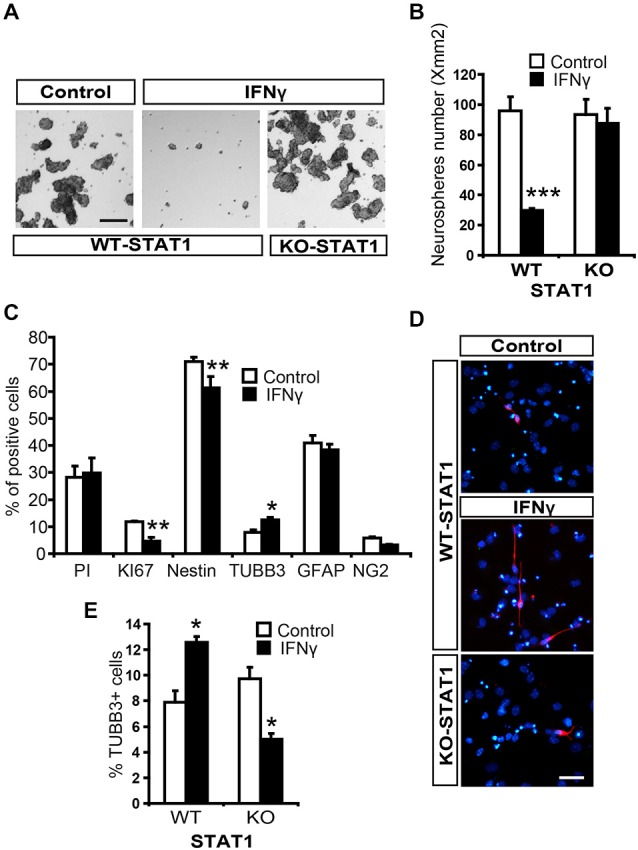
**IFNγ regulates proliferation and differentiation of NSCs from SVZ through STAT1. (A)** Brightfield pictures showing how the number of secondary neurospheres was dramatically reduced by IFNγ and that STAT1 prevents cytokine actions. **(B)** Histogram showing how IFNγ impedes neurosphere formation through STAT1 (*n* = 5). **(C)** Histogram demostrating the presence of neural cells after 7-day NSCs culture differentiation. The presence of IFNγ did not affect viability (PI incorporation); whereas the number of KI67+ and original Nestin+ cells decreased, and the number of committed neuroblasts (TUBB3+ cells) increased, without affecting oligodendroglial differentiation (NG2+ cells) or the number of GFAP+ cells (*n* = 5). **(D)** Photographs illustrating the increased neurogenesis induced by IFNγ through STAT1. IFNγ induced a higher neuroblast (red) differentiation, which reverted in the absence of STAT1. Hoechst (blue) stained all nuclei (*n* = 5). **(E)** Histogram demonstrating the presence of neuroblasts (TUBB3+ cells) originated from neural 7-day NSC culture differentiation. IFNγ improves neuronal differentiation in WT cultures but not in STAT1 deficient cells (*n* = 5) in which a slightly reduction in neuroblasts was detected. Scale bar: **(A)**, 100 μm and **(D)**, 40 μm. Data are represented as mean ± SEM. *p < 0.05; **p < 0.01; ***p < 0.001.

To explore the effects of IFNγ on NSCs, we performed neurosphere assays and assessed the effects of this molecule on the stem cell population (see “Materials and Methods” Section, Ferron et al., 2007). The number of secondary neurospheres formed was severely decreased by the presence of IFNγ (Figures 4A,B). Cell viability was evaluated using Trypan blue and no differences were observed between treated and untreated neurospheres, thus showing that survival was not affected by this cytokine (data not shown). It was not possible to determine the impact of IFNγ on self-renewal, since secondary neurospheres previously treated with IFNγ were unable to form tertiary neurospheres, indicating that the antiproliferative effect it exerts is irreversible.

To study the differentiation of NSCs from the SVZ, tertiary neurospheres were disaggregated and cultured in differentiating conditions. The presence of IFNγ decreased the proportion of NSCs in the culture (Nestin+ cells, undifferentiated) while neurogenesis was enhanced (TUBB3+ cells) and glial differentiation was maintained (Figures 4C,D). We then explored the operative effects of IFNγ on neuronal differentiation in the absence of STAT1, using cells from STAT1-deficient mice. IFNγ did not exert its effects in the absence of STAT1 (Figures 4D,E). Surprisingly, IFNγ slightly decreased the proportion of TUBB3+ cells in the STAT1 KO cultures (Figure 4E). This result indicates that, besides its major effects mediated through STAT1, IFNγ could activate other pathways in this specific *in vitro* context.

Altogether, these results suggest that IFNγ acts on neural SVZ progenitors and plays a role in the regulation of progenitor proliferation and neuronal differentiation.

### IFNγ Decreases Progenitor Proliferation and Enhances Neuronal Differentiation *In Vivo*

**Figure 5:**
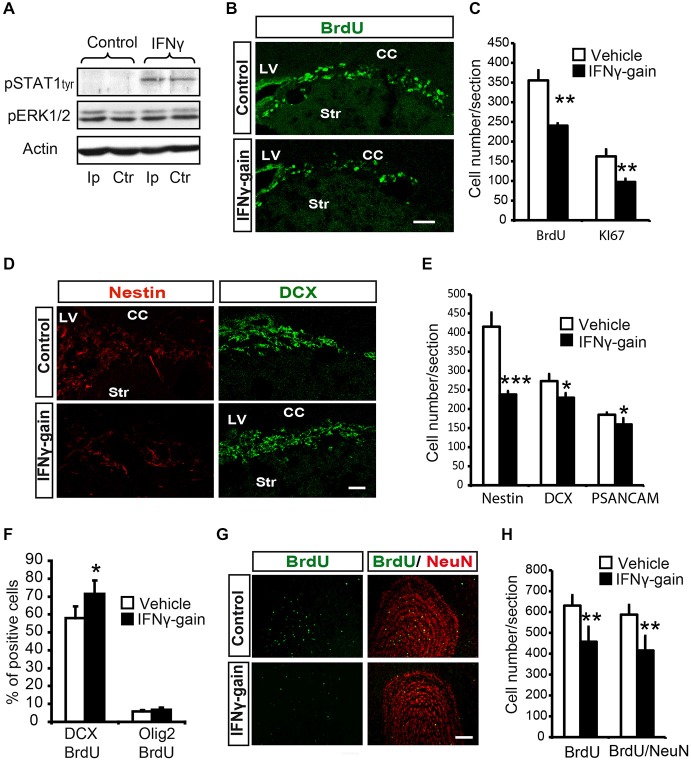
**IFNγ modulates proliferation and neuronal differentiation in adult SVZ and causes a final neurogenesis impairment in the olfactory bulb(OB) *in vivo*. (A)** Phosphorylation of STAT1 in the ipsilateral (Ip) and contralateral (ctr) SVZ niche of adult mice 30 min after they received an intracerebroventricular (ICV) injection of IFNγ (1 μl of 50 ng/ml) (*n* = 3). **(B)** Pictures illustrating the decrease of Ki67 in dorsal SVZ after *in vivo* gain of function of IFNγ (*n* = 4). **(C)** Histogram representing the total number of positive cells in the dorsal SVZ. BrdU+ cells and Ki67+ cells decreased in IFNγ-gain animals. **(D)** Pictures of dorsal SVZ showing substantial decrease in Nestin+ cells and mild differences in DCX+ cells of IFNγ-gain animals. **(E)** Quantification summarizes the robust decrease in Nestin+ progenitor cells and the mild reduction in neuroblast populations (DCX+ or PSA-NCAM+) in the SVZ niche of IFNγ-treated animals (*n* = 6). **(F)** Histograms summarizing cellular fate determination (expressed as percentage) in animals injected with BrdU at time 0 and with a pump implanted for 4d. Neuronal fate determination (BrdU+/DCX+) was significantly increased and glial production (BrdU+/Olig2+) was unaltered by IFNγ (*n* = 5). **(G)** Pictures of the OB showing the presence of BrdU+, NeuN+ and BrdU+/NeuN+ labeled cells in IFNγ-gain mice. The number of newborn cells (BrdU) and neurons (BrdU/NeuN) were reduced in the animals in which IFNγ activity was induced. **(H)** Histograms summarizing the quantification of total number of newborn cells and neurons per section in the OB of controls and IFNγ-gain animals (*n* = 5). The number of newborn cells (BrdU+) and neurons (BrdU+/NeuN+) was reduced in the presence of IFNγ. Scale bar: **(B, D)** and **(G)** 100 μm. Data are represented as mean ± SEM. *p ≤ 0.05; **p ≤ 0.01; ***p ≤ 0.01.

A single intracerebroventricular (ICV) administration of IFNγ into the LV of the mice induced STAT1Tyr705 phosphorylation in the SVZ niche of both hemispheres, contralateral and ipsilateral to the injection site (Figure 5A). This indicates that, as in the *in vitro* case, IFNγ activates STAT1 *in vivo*.

As described previously, we developed and *in vivo* gain-of-function model (Pérez-Asensio et al., 2013) to elucidate the physiological role of IFNγ. The LV (right hemisphere) of adult mouse brains were infused with very low doses of cytokine (IFNγ-gain), BrdU was administered to monitor newly born cells, and histological studies were carried out on the left (contralateral) hemisphere (for detailed procedure see Pérez-Asensio et al., 2013). The expression levels of astroglial and microglial markers, such as GFAP and ionized calcium-binding adapter molecule 1 (Iba-1), were similar for control and IFNγ-gain-of function animals (data not shown), indicating that cytokine administration using this experimental protocol did not induce a glial reaction, as we previously reported (Pérez-Asensio et al., 2013).

First, histological exploration of the whole SVZ of IFNγ-treated mice showed that both proliferating (BrdU+, 357 ± 25.8 vs. 240.5 ± 5.9) and cycling (Ki67+, 162.5 ± 9.4 vs. 97.4 ± 8.9; Figures 5B,C) cells were severely reduced in the SVZ niche in the IFNγ-gain animals, which indicates that IFNγ regulates progenitor proliferation *in vivo*.

The histological analysis indicated no changes in the total number of cells (TO-PRO3+) or cell death (see “Materials and Methods” Section) in the IFNγ-gain *in vivo* model. Moreover, analysis at the cellular level revealed a severe reduction in the number of Nestin+ cells after IFNγ treatment (Figures 5D,E). The number of committed neuroblasts, either DCX+ cells (272.9 ± 18.4 vs. 225.7 ± 12) or PSA-NCAM+ cells (184.8 ± 5.7 vs. 159.6 ± 6.1), was slightly reduced (Figures 5D,E). Since IFNγ affects proliferation, we administered a single injection of BrdU followed by 4 days of IFNγ treatment to define precisely whether IFNγ affects the phenotype diversity of original progenitors *in vivo*. Four days is the time required to detect newly formed neuroblasts in SVZ regeneration experiments (Doetsch et al., 1999). Using this experimental approach, IFNγ selectively increased the proportion of committed neuronal cells (BrdU/DCX), from 57.9 ± 6.5% to 71.5 ± 7.3%; while it did not affect the number of BrdU/Olig2 double-labeled cells (Figure 5F). In conclusion, IFNγ stimulates neurogenesis without modifying glial phenotype differentiation.

Therefore, IFNγ reduces the generation of Nestin+ cells but neuroblast numbers are maintained, since IFNγ also specifically promotes neuronal differentiation (Figure 5F). These results show that IFNγ regulates both progenitor proliferation and neuronal differentiation *in vivo*.

### IFNγ Regulates Neuronal Differentiation in the SVZ Niche, Causing a Final Modulation of Neurogenesis in the OB

Finally, we analyzed the OB, i.e., the final target area of newly generated neurons, of IFNγ gain-of-function animal models to study the ultimate repercussion of IFNγ. The experimental design consisted of BrdU administration at days 0 and 1, followed by 19 monitoring days. This waiting time is required to allow newly formed cells to become fully differentiated neurons (NeuN+) that reach the OB: the target area (Doetsch and Alvarez-Buylla, 1996). Immunoanalysis of the OB of the IFNγ-gain animals showed a reduced number of BrdU+ cells and also a decrease in the total number of newly generated neurons (BrdU/NeuN double+ cells, 584.6 ± 46.7 vs. 414.6 ± 70.1; Figures 5G,H), supporting the idea that the outcome of IFNγ actions is a reduction in final neurogenesis in the target region. This result indicates that prolonged and persistent antiproliferative actions exerted by IFNγ are not compensated for by its differentiating effects on progenitor pools.

Altogether, these results indicate that IFNγ activity participates in the control of neurogenesis in the adult SVZ by regulating progenitor proliferation and neuronal differentiation, which in the end reduces the number of neurons that regularly regenerate the OB.

### STAT1 Knockout Mice have Normal Physiological Neurogenesis but Show an Impaired Response to IFNγ Action *In Vivo*

**Figure 6:**
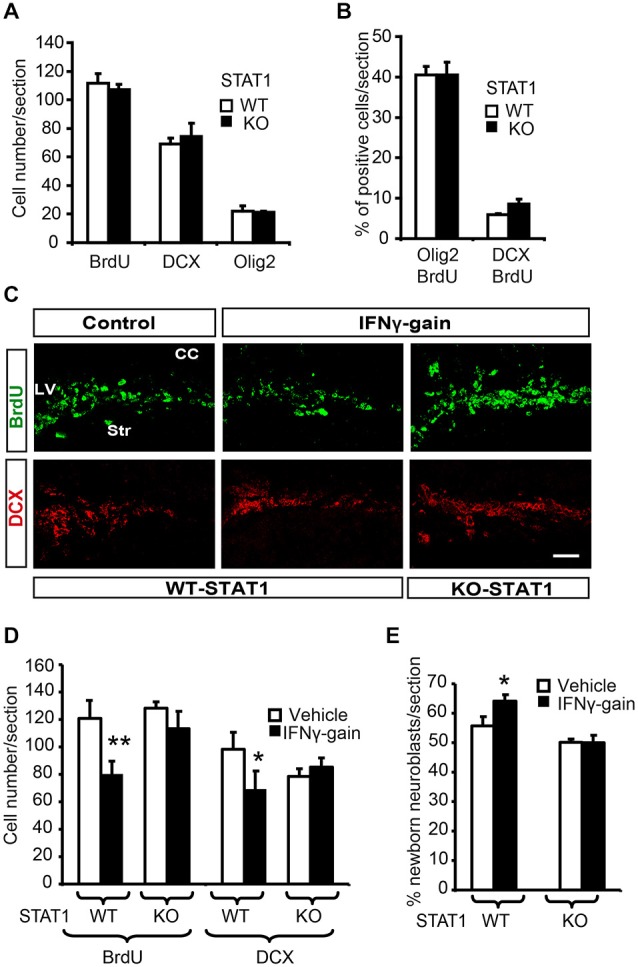
**STAT1 mediates IFNγ activities *in vivo*. (A)** Quantification summarizes how, in the absence of STAT1, proliferation (BrdU) and the expression of neuronal markers such a DCX and olig2 were unaltered. **(B)** Histogram representing a similar proportion of newborn determined neurons (BrdU+/DCX+) and glial cells in the SVZ of STAT1-KO animals that received a single BrdU 4 days before sacrifice, (*n* = 6). **(C)** Pictures of the SVZ sections illustrating that STAT1 deficiency impeded IFNγ functions in the *in vivo* model. The numbers of proliferative cells (BrdU, green) and neuroblasts (red) were unchanged in KO samples in the IFNγ-treated animals. **(D)** Quantification summarizes how IFNγ effects on proliferation (BrdU incorporation) and neuroblast populations (DCX+ cells) were abolished in the absence of STAT1 mediator (*n* = 6). **(E)** Histogram representing the impossibility of IFNγ inducing neuronal differentiation (BrdU+/DCX+ cells) in STAT1-KO animals that received a single BrdU administration 4 days before sacrifice. (*n* = 6). Scale bar: 50 μm. Data are represented as mean ± SEM. *p < 0.05; **p ≤ 0.01.

Next, the effects of IFNγ on a STAT1 KO animal were explored. First, we evaluated regular proliferation and neurogenesis in the SVZ of STAT1 KO animals. No difference was observed in the presence of proliferating cells (BrdU+), neuroblasts (DCX+) or glial progenitors (Olig2+ cells) in the SVZ of STAT1 KO animals compared with the corresponding wild-type (WT) mice (Figure 6A). In addition, *in vivo* differentiation studies also showed that neuronal and oligodrendroglial differentiation was normal in the adult SVZ niche in the absence of STAT1 (Figure 6B).

When IFNγ-gain was performed in the absence of STAT1, no effects were observed on cell proliferation or the proportion of DCX+ cells in the SVZ niche; thus indicating that IFNγ activity is mediated through STAT1 (Figures 6C,D). To further support the role of STAT1 as the IFNγ mediator, we analyzed endogenous neurogenesis in a model of IFNγ-gain in the STAT-KO mice. No differences were observed in WT vs. STAT1 KO animals when IFNγ was administered, since the number of new neurons (BrdU+/DCX+) after 4 days of BrdU administration was similar (50.1 ± 0.7 vs. 50.0 ± 1.9%, respectively; Figure 6E). No differences in the number of newly generated glial cells were observed between the two genotypes (BrdU/Olig2+; Figure 6E).

Altogether, these results prove that IFNγ, through the mediation of STAT1, participates in the regulation of adult SVZ neurogenesis by interfering in both progenitor proliferation and differentiation, which as a result modulates final neurogenesis in the target region.

## Discussion

The SVZ niche has a strategic brain location that allows a complex network of interactions between neighboring cells, the vasculature, and the CSF. This rich environment provides molecular signals that regulate cell renewal and proliferation, as well as and identity of the resident progenitors (Lois and Alvarez-Buylla, 1994; Doetsch et al., 1999; Merkle et al., 2004; Ramírez-Castillejo et al., 2006; Villeda et al., 2011; Delgado et al., 2014). Our present data demonstrate through a gain-of-function model and the use of genetically deficient mice, that IFNγ has a dual effect on SVZ progenitors mediated by STAT1. STAT1 is activated by IFNγ in Nestin+ progenitors where it acts as a potent inhibitor of proliferation. In addition, IFNγ exerts a pro-neurogenic effect on those progenitors, and this also requires STAT1 activity. Finally, *in vivo*, the balance of the two IFNγ activities leads to an important reduction in neurogenesis in the final target region: the OB. All these findings together, indicates that local levels of IFN-gamma could act as sensor of body physiology providing inputs to the SVZ niche by targeting Nestin+ progenitors in order to regulate neurogenesis. On that direction, it has been previously described that IFN (−/−) mice have a negative impact on SVZ progenitors in non-inflammatory brain (Li et al., 2010).

Our present findings identifying IFNγ as a relevant antiproliferative agent in the adult SVZ are in agreement with previously reported data showing that the addition of IFNγ to SVZ progenitor cultures inhibits neurosphere formation (Wong et al., 2004; Li et al., 2010; Turbic et al., 2011). Moreover, SVZ cells from chronically IFNγ-deficient adult mice generate more and on average larger SVZ-derived neurospheres with improved self-renewal and multipotentiality (Li et al., 2010). These observations are supported by our *in vivo* data showing that IFNγ deficiency leads to a rise in the number of mitotic BrdU-positive cells detected in SVZ animal sections, as well as a relevant increase in the percentage of newborn neurons incorporated into the OB (Li et al., 2010). The role of IFNγ in neuronal differentiation is more controversial. Here, we describe IFNγ as an important neurogenic factor *in vivo*, in consonance with data from other authors who report that IFNγ increases neurogenesis in a variety of progenitor cultures of different origins (Wong et al., 2004; Song et al., 2005; Zahir et al., 2009; Turbic et al., 2011; Walter et al., 2011). In contrast, Li et al., 2010) observed that primary neurospheres from IFNγ-deficient mice showed higher numbers of both oligodendrocytes and neuroblasts; and the addition of IFNγ to these IFNγ-deficient cultures almost eradicated the TUBB3+ cells, which reached extremely low values compared with WT cultures. All these apparent discrepancies between the reported *in vitro* data may be explained by different culture origins and conditions, including cell genetics and origin, assay conditions, type of IFNγ treatment, and the number of passages of neurospheres. In addition, the cited studies did not report cell viability and proliferation, which can be important for combining and evaluating final outcomes in terms of cell production. Here we demonstrate, for the first time *in vivo*, in the SVZ niche, that IFNγ increases neuronal differentiation without affecting glial differentiation, and that this action is mediated by STAT1. Accordingly, under pathological conditions in animal models of neurodegeneration, IFNγ enhances neurogenesis in the adult dentate gyrus (Baron et al., 2008; Mastrangelo et al., 2009). In spite of the effect of IFNγ in promoting neuronal differentiation, our *in vivo* data show important impairment of neurogenesis in the final target region, the OB, of mice treated with IFNγ. Therefore, the pro-neurogenic activity of IFNγ could not finally overcome the strong antiproliferative effect of this cytokine, and as a result of this, overall neurogenesis in the OB was reduced.

The IFNGR signals through the activation of the JAK/STAT pathway in several systems. This canonical pathway involves the activation of JAKs, and phosphorylation of STAT proteins that translocate into the nucleus where STATs act as transcription factors (Li, 2008). IFNγ-induced STAT1 activation by JAK adaptors is a key element mediating its well-known pro-inflammatory properties in several systems including the nervous system (Maher et al., 2007; Hu and Ivashkiv, 2009; Saha et al., 2010). During mammalian cerebral development, the NSCs present in germinal regions give rise to successive waves of neurons, followed by oligodendrocytes and astrocytes, in a process coordinated by bone morphogenetic proteins (BMPs) and some cytokines (Bonni et al., 1997; Mehler et al., 2000). Cytokine activation of JAK-STAT during development promotes the differentiation of precursors along a glial lineage and its activity is inhibited during neurogenesis to prevent premature astrocyte differentiation (Mayer et al., 1994; Bonni et al., 1997; Nakashima et al., 1999; He et al., 2005; Li, 2008; Kanski et al., 2014). In the present study, we found that IFNγ activates Tyr phosphorylation of STAT1 exclusively in Nestin+ progenitors, and this activation is at least partially mediated by JAK2. In contrast to developmental stages, STAT1 activation in adulthood specifically promotes neurogenesis without interfering with glial differentiation.

Here, we report endogenous effects of IFNγ on adult neural progenitors in the normal brain. IFNγ has a dual effect: it reduces proliferation and promotes neuronal differentiation. Moreover, we demonstrate that STAT1 is activated by IFNγ in Nestin+ progenitors and mediates both effects induced by this cytokine *in vivo*. The robust antiproliferative activity of IFNγ overcomes the pro-neuronal activity, and the final impact is an impairment of neurogenesis in the OB.

## Author Contributions

LP, MB and RM performed experiments and analyzed some data. AMP contributed to the set-up of the experiments and was involved in drafting and revising the manuscript. EP designed and performed experiments, supervised the study, analyzed the data and wrote the manuscript. All the authors agree that all the questions related to the accuracy or integrity of the work have been appropriately researched and resolved, giving their final approval of the version to be published.

## Conflict of Interest Statement

The authors declare that the research was conducted in the absence of any commercial or financial relationships that could be construed as a potential conflict of interest.
